# The Link4Health study to evaluate the effectiveness of a combination intervention strategy for linkage to and retention in HIV care in Swaziland: protocol for a cluster randomized trial

**DOI:** 10.1186/s13012-015-0291-4

**Published:** 2015-07-19

**Authors:** Margaret L. McNairy, Averie B. Gachuhi, Matthew R. Lamb, Harriet Nuwagaba-Biribonwoha, Sean Burke, Peter Ehrenkranz, Sikhathele Mazibuko, Ruben Sahabo, Neena M. Philip, Velephi Okello, Wafaa M. El-Sadr

**Affiliations:** ICAP, Mailman School of Public Health, Columbia University, 722 West 168th Street, New York, NY 10032 USA; Weill Cornell Medical College, New York, NY USA; Centers for Disease Control, Mbanane, Swaziland; Ministry of Health, Kingdom of Swaziland, Mbabane, Swaziland; Department of Epidemiology, Mailman School of Public Health, Columbia University, New York, NY USA

**Keywords:** HIV, Linkage, Retention

## Abstract

**Background:**

Gaps in the HIV care continuum contribute to suboptimal individual health outcomes and increased risk of HIV transmission at the population level. Implementation science studies are needed to evaluate clinic-based interventions aimed at improving retention of patients across the continuum.

**Methods/design:**

Link4Health uses an unblended cluster site-randomized design to evaluate the effectiveness of a combination intervention strategy (CIS) as compared to standard of care on linkage to and retention in care among HIV-diagnosed adults in Swaziland. The CIS intervention targets a multiplicity of structural, behavioral, and biomedical barriers through five interventions: (1) point-of-care CD4 testing at time of HIV testing, (2) accelerated antiretroviral therapy (ART) initiation for eligible patients, (3) mobile phone appointment reminders, (4) care and prevention packages, and (5) non-cash financial incentives for linkage and retention. The unit of randomization is a network of HIV clinics inclusive of a secondary facility coupled with an affiliated primary facility. Ten study units were randomized based on implementing partner, geographic location, and historic volume of HIV patients. Target enrollment was 2200 individuals, each to be followed for 12 months. Eligibility criteria includes HIV-positive test, age >18 years, willing to receive HIV care at a clinic in the study unit and consent to study procedures. Exclusion criteria included previous HIV care in the past 6 months, planning to leave the community, and current pregnancy.

The primary study outcome is linkage within 1 month and retention at 12 months after testing HIV positive. Secondary outcomes include viral load suppression at 12 months, time to ART eligibility and initiation, participant acceptability, and cost-effectiveness. The trial status is that study enrollment is complete and follow-up procedures are ongoing.

**Discussion:**

Link4Health evaluates a novel and pragmatic combination intervention strategy to improve linkage to and retention in care among adults with HIV in Swaziland. If the strategy is found to be effective, this study has the potential to inform HIV service delivery in resource-limited settings.

**Trial registration:**

Clinicaltrials.gov NCT01904994

## Background

Patient engagement and retention in the HIV care continuum is essential to optimize health outcomes for the individual and to reduce HIV transmission to others [1–3]. Linkage from HIV testing to HIV care is the first step in the continuum and is essential for accessing appropriate HIV care and treatment interventions [4]. Retention in ongoing HIV care is needed for clinical and laboratory monitoring of disease progression, assessment for eligibility for antiretroviral therapy (ART), initiation of ART when eligible, effective prevention of mother-to-child transmission (PMTCT), and provision of support and prevention [4–8]. Multiple studies conducted in sub-Saharan Africa report high attrition from testing to linkage to care and suboptimal retention in ongoing care [9–18].

While several studies have evaluated the effect of an individual intervention on one step in the HIV care continuum, few have evaluated a combination approach that includes multiple interventions bundled into a coherent strategy that would target numerous barriers along the continuum [19]. A combination approach must include structural, biomedical, and behavioral interventions that address specific barriers reported in the literature [14, 16, 20–30]. Additionally, there is the need for implementation science research to evaluate proposed combination approaches in a “real-life” context to provide pragmatic information on uptake of interventions, feasibility, and acceptability [31, 32].

Swaziland is a small country in Southern Africa with the world’s most severe HIV epidemic, and HIV-related illness is the leading cause of death in the country. It has a population of 1.1 million persons, an estimated adult (age 18–49 years) HIV prevalence of 31 % and an estimated incidence of 2.4 % [33–35]. The country has made impressive advances in responding to the epidemic. In 2011 at the time of this study start, 93,295 adults had cumulatively been initiated on ART across the country, and by 2013, this number increased to 133,420 [36]. Nevertheless, rates of linkage to care and retention at 12 months after ART initiation remain suboptimal [37, 38]. Swaziland’s Ministry of Health is committed to improving retention along the HIV care continuum in order to decrease the impact of HIV on morbidity and mortality as well as to decrease the alarming incidence rate of HIV.

We describe the design of Link4Health, an implementation science study that aims to assess the effectiveness of a combination strategy comprised of five evidence-based interventions designed to improve linkage to and retention in care among adults newly tested HIV positive in Swaziland.

### Evidence for selected interventions

Each intervention used in the combination strategy was selected based on being practical and having prior evidence supporting its use for improving linkage to and/or retention in HIV care in a resource-limited setting.

The first intervention, point-of-care CD4+ count testing, when done at the time of receipt of result of an HIV-positive test, reduces the number of visits needed to identify patients who are eligible for ART from multiple visits to a single visit. As such, it addresses a structural barrier implicit in the need for multiple visits previously required prior to ART initiation. Several studies have reported higher linkage rates with point-of-care CD4+ count testing as compared to traditional CD4+ count testing [39–42]. In addition, provision of a CD4+ count result to a patient at the time of HIV diagnosis may serve to motivate faster linkage to care, especially for a patient who is found to be eligible for treatment.

The second intervention in the combined strategy, accelerated ART initiation for eligible patients, reduces the number of clinic visits required prior to initiation among eligible patients. This is important as high mortality has been noted among HIV-infected individuals who fail to engage in care, particularly among those with advanced disease [12, 43–45].

The third intervention in the combined strategy is the use of short message service (SMS) reminders for clinic appointments. SMS reminders have been used in HIV care and other chronic disease management to improve health communication and patient adherence [46–54]. The fourth intervention is a basic care and prevention package which provides counseling and health commodities such as condoms, pillboxes, and soap with the goal of improving health prevention and health outcomes [55–59]. A similar intervention was evaluated in Uganda and was associated with high rates of cotrimoxazole use, condom use, and HIV testing of family members [55]. The fifth intervention is non-cash financial incentives. There has been great interest in the use of financial incentives as a structural intervention to achieve positive health behaviors [60–66]. In this study, the non-cash financial incentives are targeted at mitigating the costs of care which include transport costs and opportunity costs, such as lost wages and/or need to obtain childcare.

## Methods

### Study design

**Fig. 1:**
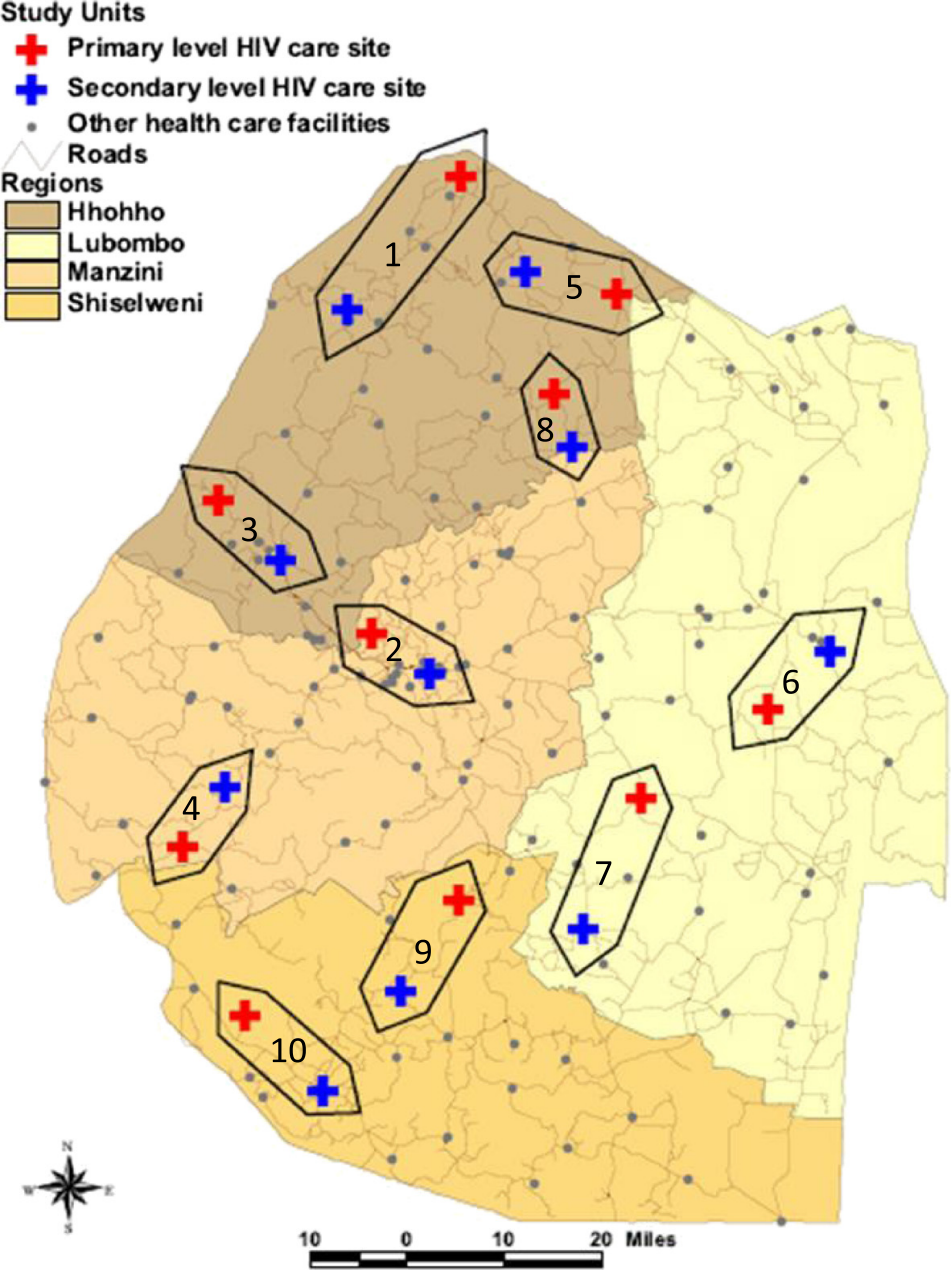
Link4Health study units

Link4Health is a cluster site-randomized trial. The unit of randomization is a network of secondary HIV clinics paired with an affiliated primary-level HIV clinic, which is referred to as a study unit (Fig. 1). The study unit reflects the ongoing process of decentralization of HIV care from larger facilities to primary-level facilities in Swaziland [38]. Ten study units were selected from the 11 existing secondary facilities in the country. The largest primary-level facility affiliated with each selected secondary facility was selected based on HIV program size (i.e., number of HIV patients in follow-up at the site based on 18-month historic data, with special attention given to the most recent 6 months). Study units were randomized to the combination intervention strategy (CIS) or standard of care (SOC) strategy using matched-pair randomization balanced by the following factors: facility location (rural, urban), expected number of adults enrolled in HIV care at each facility per month, and implementing partner supporting the facility (Table 1). Sites within matched pairs were randomized to receive either CIS or SOC by generating a random integer using Microsoft Excel, taking the alphabetically first site name if the random integer drawn was 1, and the alphabetically second site name if the random integer drawn was 2.

**Table 1:** Study unit matched pairs

Matched pair	Study unit	Study arm	Implementing partner	Location
1	1	SOC	Partner A	Urban
	2	CIS	Partner A	Urban
2	3	SOC	Partner A	Urban
	4	CIS	Partner A	Urban
3	5	SOC	Partner A	Rural
	6	CIS	Partner A	Rural
4	7	SOC	Partner A	Rural
	8	CIS	Partner A	Rural
5	9	CIS	Partner B	Urban
	10	SOC	Partner B	Urban

*SOC* standard of care, *CIS* combination intervention strategy

Individuals who tested HIV positive at HIV testing sites located at the study unit received post-test counseling and were referred to the study by clinic staff. Thereafter, the study research staff provided an overview of the study, conducted eligibility screening, and conducted informed consent procedures. Study eligibility criteria included the following: HIV-positive test, ≥18 years of age, willing to receive HIV care at a clinic in the study unit, and consent to study procedures. Study exclusion criteria included the following: planning on leaving the community during the study period for more than 6 months, enrolled in HIV care and/or initiated ART in the past 6 months, currently on ART, does not speak English or SiSwati, or current pregnancy.

### Study objectives and outcomes

The primary study objective was to evaluate the effectiveness of the CIS compared to SOC on the combined outcome of linkage to HIV care within 1 month of an HIV-positive test and retention in care at 12 months among adults diagnosed with HIV. Linkage to care was defined by at least one visit to HIV clinic with completion of an intake assessment including medical history and physical exam. Retention in care at 12 months after HIV testing was defined as a clinic visit within 90 days prior to the end of the study follow-up period.

The secondary objectives included the evaluation of the effectiveness of CIS compared to SOC with regard to the following: linkage to care within 1 month after testing HIV positive, retention in care at 12 months after testing HIV positive, time to ART eligibility and ART initiation, viral load suppression at 12 months after HIV testing, disease progression, patient acceptability, and cost-effectiveness. Additionally, the effect of sociodemographic and clinical determinants on key study outcomes was to be assessed.

### Study interventions

#### Standard of care

At study units assigned to the SOC arm of the study, participants were managed as per country guidelines. After testing HIV positive, individuals received post-test counseling and were referred to an HIV clinic using a national referral form that documents the patient’s identification and HIV status. Patients who linked to HIV care received clinical and laboratory assessments for the determination of ART eligibility, including CD4+ count testing and hematology and chemistry tests, and were instructed to return to the clinic in 1 to 2 weeks to receive their results. Upon return, those found eligible for ART initiation received three counseling sessions. The time interval from eligibility determination to ART initiation typically has been 1 to 2 months. All patients were given cotrimoxazole prophylaxis and those not eligible for ART were instructed to return every 3 months to the clinic, while those who initiated ART returned to the clinic every month for 6 months, and then every 3 months if they were stable on treatment. Peer counselors were encouraged to call patients within 7 days of a missed appointment; however, these procedures were often not consistently applied. Condoms and informational materials were available for all patients at HIV clinic visits.

#### Combination intervention strategy

Participants at study units assigned to the CIS study arm received five evidence-based interventions in addition to SOC interventions. The five interventions include the following: (1) point-of-care CD4+ count testing on the same day as HIV diagnosis, (2) accelerated ART initiation for eligible patients with CD4+ count <350 cells/μL, the prevailing ART eligibility threshold during the conduct of the study, (3) mobile phone appointment reminders, (4) care and prevention bags, and (5) non-cash financial incentives for linkage and retention. Table 2 shows the interventions included in the study, the type of barrier addressed, and the target step in the HIV care continuum. Clinic staff at study units assigned to CIS were trained on each of these interventions and the importance of their provision to all study participants at their site.

**Table 2:** Study interventions, type of intervention, and target step in the HIV care continuum

Intervention	Standard of care (SOC)	Combination intervention strategy (CIS)	Type of intervention	Step targeted in HIV care continuum
Point-of-care CD4+ count testing	• Point-of-care CD4 assays available in some primary care clinics and some secondary health centers/hospitals for patients enrolled in HIV care but only used once a patient has linked to care	• Point-of-care CD4 assays at the HIV testing site at the time of HIV testing	Structural and biomedical	Linkage and ART initiation
		• Turnaround time immediate		
	• All clinics have CD4 (Cyflow, FACS Caliber) availability after linkage to HIV care in the clinic or lab			
	• Turnaround time approximately 2 weeks			
Accelerated ART initiation	• ART initiation per national guidelines for patients with CD4 ≤350 cells/μL or WHO stage III/VI	• Accelerated ART initiation for patients with point-of-care CD4 ≤350 cells/μL within 1 week from testing	Structural and biomedical	ART initiation and retention
	• Requires 3 counseling sessions and receipt of baseline lab tests	• 2 counseling sessions (one at the time of HIV testing and another at the first HIV clinic visit), and collection of blood for other baseline lab tests, but ART initiation prior to return of results for patients who do not meet the criteria for waiting for receipt of lab results prior to ART initiation		
	• Initiation 2 weeks–1 month from testing			
Cellular appointment reminders	• Telephone call within 7 days of missed appointment for ART patients only	• Short message service (SMS) (or voice if illiterate) appointment reminders 1 day prior to each scheduled appointment	Behavioral	Linkage and retention
		• SMS (or voice if illiterate) reminder within 7 days after a missed appointment		
Care and prevention services	• Cotrimoxazole prescribed for all patients once enrolled in HIV care	• Basic care and prevention package provided approximately every 3 months. Package includes condoms, soap, cotrimoxazole, pillbox, and pictorial education about the use of materials and HIV, such as family testing tools and information	Biomedical and behavioral	Retention
	• Condoms available			
Non-cash financial incentive	• None	• Non-cash financial incentive (mobile airtime) provided for linkage within 1 month of testing and retention at 6 and 12 months	Structural	Linkage and retention

1. *Point-of-care CD4+ count testing*. Each participant at sites randomized to CIS received a point-of-care CD4+ count test immediately after testing HIV positive. The CD4+ count test was conducted by the study staff and took approximately 20 min to process. CD4+ count results and their implications were provided to the participant, and a copy was placed in the patient’s national referral form for HIV care. Regardless of the CD4+ count result, all participants were encouraged to link to HIV care within 1 week of receiving the results.
2. *Accelerated ART initiation for eligible participants*. This intervention streamlined the preparatory procedures required for ART initiation by consolidating the ART counseling sessions into two sessions, as compared to three in standard of care, in order to enable ART initiation within 1 week of diagnosis. At sites assigned to CIS, the first ART preparatory counseling session was given at the time of receiving the point-of-care CD4+ count result at the HIV test site. The second session was given by the clinic staff at the HIV care site, at the first visit upon linkage to care by the participant. Clinic staff collected samples for the required baseline laboratory tests per national guidelines. Clinicians were encouraged to initiate ART at this first care visit, rather than awaiting the results of these laboratory tests in participants with no comorbid conditions. For participants that needed further counseling and/or clinical assessments, initiation of ART was done at the discretion of the clinician. Participants who initiated ART received a 2-week supply of medications per national guidelines. Baseline laboratory results were reviewed by the study staff with the clinic staff soon after availability, and if any abnormality was noted, participants were contacted to return to the clinic. After initiating ART, all follow-up visits followed national guidelines, similar to the standard of care procedures.
3. *Mobile phone appointment reminders*. All participants at sites assigned to CIS received appointment reminders via mobile phone SMS, or voice message reminders for illiterate participants, to encourage linkage to care and completion of scheduled clinic visits. Reminders were sent to the participant’s phone or on a friend or relative’s phone per participant preference. The reminders did not refer to HIV or reveal any personal information. An example message is: “Good day – This is a reminder about your visit at [health facility] tomorrow. We look forward to seeing you then*.*” SMS were sent from a central server 1 day prior to scheduled clinic visit date for all participants. For participants who missed appointments, a text message was sent within 7 days to encourage the participant to return to the clinic.
4. *Basic care and prevention package*. All participants at sites assigned to CIS received a package of commodities and informational materials upon linkage to care and once every 3 months thereafter during the study period. This package was designed to offer an opportunity to have further counseling on HIV care through provision of commodities that were deemed of value by the patients. Commodities in the package included condoms, a pillbox, soap, toothbrush and paste, cotrimoxazole tablets, and an appointment calendar. The health counseling that accompanied each package used existing national health information and educational materials (i.e., pamphlets, pictures) that focused on medication adherence, family planning, tuberculosis screening and cough hygiene, and nutrition.
5. *Non-cash financial incentive (FI)*. All participants at sites assigned to CIS who linked to care within 1 month of HIV testing and those who completed HIV care clinic visits at 6 and 12 months after HIV testing received a non-cash FI in the form of prepaid mobile phone airtime vouchers. Each FI consisted of 80 Swaziland Emalangeni ($8–10), an amount selected based on the estimated cost of traveling to the clinic and with consultation with key stakeholders in the country.

### Data collection

All participants completed a baseline interview, at the time of study enrollment, which included information on sociodemographics, HIV history, barriers to care, travel time to clinic, depression, social and family support, and HIV-related knowledge. Follow-up interviews were conducted at home at 1 and 12 months after enrollment to collect information on changes in sociodemographic characteristics, utilization of HIV services including self-reported linkage to care and visit completion, and acceptability of the study interventions. A CD4+ cell count was obtained at 12 months. Additional clinical and laboratory data were extracted from paper-based patient medical charts. Facility assessment surveys were conducted at the beginning of the study and every 6 months to document any changes in clinical services that occurred during the study period that may influence study outcomes. Costs of delivering the SOC and CIS were estimated based on the cost of outpatient HIV care, the cost of hospitalization, and the cost of delivering each intervention component.

### Sample size and power calculations

We estimated that 35 % of participants in the SOC study arm would achieve the combined primary outcome (assuming 50 % link to HIV care within 1 month and 70 % of those linking within 1 month are retained 12 months after study enrollment). We estimated that approximately 2750 adults would be eligible for study enrollment based on historic testing volume, the proportion testing HIV positive at the study units from 2010 to 2011. Assuming 80 % of eligible individuals consent to enroll in the study, we estimated an average enrollment of 220 participants per study unit or 2200 in total with 1100 per study arm.

With this sample size and five study units per study arm, we estimated the minimum difference in the primary outcome we could detect with 80 % power using a two-sided Farrington and Manning Likelihood Score Test [19], assuming an interclass correlation coefficient of 0.05. Estimates were made using the Power and Sample Size (Pass 8.0) software program. With these assumptions, the study has 80 % power to detect an absolute increase of 20 % or more in the CIS versus SOC arm on the primary outcome of linkage within 1 month and retention at 12 months.

### Statistical methods

An intent-to-treat analysis was planned to compare the relative risk of achieving the primary outcome of linkage to care within 1 month and retention in care at 12 months after HIV testing. The effectiveness of CIS compared to SOC will also be assessed on secondary outcomes including the following: linkage to care within 1 month; retention 12 months after HIV diagnosis; and time from ART eligibility to ART initiation, viral load suppression 12 months after HIV diagnosis, disease progression, and cost-effectiveness. Sub-distribution hazard models, treating death as a competing risk for ART initiation, are to be used to compare the time from eligibility to initiation of ART between participants receiving CIS and SOC, with participants transferring or becoming lost to follow-up after ART eligibility but before initiation censored at their date of transfer or their last recorded visit. Robust sandwich estimates of variance will be used to account for correlation within study units. Sociodemographic and clinical determinants of the primary and secondary outcomes are to be analyzed to examine whether the CIS differentially impacts outcomes within strata by sociodemographic and clinical characteristics (e.g., age, sex, HIV disease stage, CD4+ count, tuberculosis, and pregnancy status). We will conduct covariate-adjusted generalized linear mixed models if important differences are observed between study arms in order to assess the degree to which these differences influence outcomes. For cost-effectiveness analysis, a model will be created to estimate the effect of CIS on health benefits and costs. Differences in life expectancy, quality-adjusted life years, and infections averted between CIS and SOC arms will be estimated using inputs from the study.

### Ethical considerations and study guidance

This study was approved by the institutional review boards at Columbia University, the US Centers for Disease Control and Prevention, and the Swaziland Scientific and Ethics Committee.

An advisory board was established to guide and monitor the study. This included representatives from the Ministry of Health, implementing partners and other stakeholders. The board meets quarterly and reviews challenges on clinical implementation of procedures, refinement of interventions, and discussion of the potential influence of study findings on HIV program design and planning.

## Trial status

The study commenced enrollment in August 2013 and has completed enrollment of participants. The study is currently completing 12-month follow-up procedures including questionnaires, laboratory tests, and medical record abstraction.

## Discussion

The Link4Health study leverages a combination of evidence-based interventions, which target multiple known barriers hindering linkage to and retention in HIV care. Aligned with the goals of implementation science, this study evaluates the combination strategy in a real-life clinical setting in Swaziland. The interventions were adapted to fit the unique characteristics of this setting including the health system structure, norms of practice, physical space, and available staffing at the health facilities. With such an approach, the goal is to obtain generalizable knowledge about the feasibility and effectiveness of implementing such a strategy, which could be applied in a range of settings with similar implementation contexts.

We selected a site-randomized study design as it is conducive to the implementation science approach in which study interventions are delivered at the clinic level. It is deemed more feasible for clinic staff at a site to provide all their patients with the same package of interventions, versus the situation in individual-randomized design with the complexity entailed in staff providing one patient with one intervention strategy versus another. In addition, while there is yet no evidence to suggest that the experimental intervention in this study will be superior to standard of care, individual randomization may have resulted in individuals randomized to that arm to perceive themselves as receiving an inferior strategy which may influence their health-seeking behaviors and ultimately the key study endpoints of linkage and retention.

The study design has several strengths. The study includes all regions in Swaziland, and study procedures largely mimic the HIV care structure and processes in the country. The study units took into account a common pattern of health facility structure noted in sub-Saharan Africa with smaller facilities linked to a larger facility. Eligibility criteria were selected to be broad with the aim to include as many patients as possible for generalizability. Accelerated ART was administered by clinic staff, rather than study staff, requiring minor adjustments in the traditional clinic procedures and patient flow. The cost-effectiveness analysis will help policy makers have practical budget information to guide decision-making about the intervention.

Limitations of the study design included the evaluation of retention at 12 months, which would not be sufficient to evaluate the longer-term effect of the intervention. Additionally, Swaziland has a limited number of study units, which reduces the ability to completely control for cluster overlap or provide alternate study units should a study unit halt HIV services. Finally, the study design does not allow for evaluation of the effectiveness of each component intervention in the CIS which would require a study of substantially larger size. However, results from this study can be compared to other studies in Swaziland evaluating different individual interventions aimed at improving linkage and retention in HIV care.

## Conclusion

The Link4Health study aims to improve linkage of HIV-positive patients to care and subsequently their retention in care. These two elements in the HIV care continuum are acknowledged to be of profound importance in achieving the impact of effective HIV programming on patient outcomes and reduced transmission at the population level. With the largest HIV burden in the world, Swaziland could greatly benefit from identification of an effective strategy for linkage and retention in its efforts to control its HIV epidemic. If shown to be effective, the combination intervention strategy may also serve to enhance the quality of the HIV care continuum in other countries in sub-Saharan Africa and beyond.
